# Endoscopic Pseudo‐dilation of Esophagogastric Junction During Peroral Endoscopic Myotomy: (With Video)

**DOI:** 10.1002/deo2.70360

**Published:** 2026-05-31

**Authors:** Kei Ushikubo, Haruhiro Inoue, Ippei Tanaka, Nikko Raymundo, Kazuki Yamamoto, Yuta Tamaru, Kohei Shigeta, Satoshi Abiko, Yohei Nishikawa, Mayo Tanabe, Manabu Onimaru, Koji Otuka, Takayoshi Ito, Noboru Yokoyama

**Affiliations:** ^1^ Digestive Disease Center Showa Medical University Koto Toyosu Hospital Tokyo Japan; ^2^ Institute of Digestive and Liver Diseases St. Luke's Medical Center Global City Philippines; ^3^ Gastroenterology and Hepatology Hokkaido University Hospital Hokkaido Japan

**Keywords:** endoscopy, esophageal achalasia, esophageal motility disorders, lower esophageal sphincter, myotomy

## Abstract

**Background and Aim:**

During peroral endoscopic myotomy (POEM) for achalasia, endoscopic esophagogastric junction (EGJ) opening is generally considered a sign of successful myotomy. However, in clinical practice, the EGJ may appear to open immediately after submucosal tunneling, even before myotomy is performed. We defined this phenomenon as pseudo‐dilation and evaluated changes in EGJ appearance during POEM.

**Methods:**

This retrospective study included 65 consecutive patients who underwent POEM between February and June 2025. Endoscopic images obtained at three stages—before POEM, after submucosal tunneling, and after myotomy—were evaluated using a three‐tier Endoscopic Junctional Dilation (EJD) grade. Differences in EJD grade across procedural stages were analyzed using the Friedman test, with post hoc Wilcoxon signed‐rank tests and Bonferroni correction. The Bickenböller statistic was used as a complementary analysis. Two certified endoscopists independently evaluated all images, and interobserver agreement was assessed using Cohen's κ and weighted κ.

**Results:**

The EJD grade increased significantly after submucosal tunneling compared with before POEM (*p* < 0.001). A significant difference was observed between the post‐tunneling and post‐myotomy stages (*p* = 0.002); however, most patients showed no change in EJD grade, and the overall distribution of grades remained unchanged (*p* = 0.124). Overall, pseudo‐dilation occurred in 95.4% of patients. Interobserver agreement in EJD grade assessment was substantial to perfect across all procedural stages (κ = 0.889; weighted κ = 0.940).

**Conclusion:**

Pseudo‐dilation of the EGJ was commonly observed immediately after submucosal tunneling during POEM.

**Trial Registration:**

N/A.

## 1 Background and Aim

Peroral endoscopic myotomy (POEM) has become an established, minimally invasive treatment for esophageal achalasia [1, 2, 3, 4]. Based on the principles of Heller–Dor myotomy, POEM requires adequate division of both the esophageal and gastric muscular layers, and approximately 2–3 cm of gastric‐side myotomy is generally recommended [1].

Insufficient gastric myotomy may result in reduced therapeutic efficacy, highlighting the importance of accurately assessing the distal extent of the gastric‐side myotomy [5]. Several intraoperative techniques have been proposed to objectively assess procedural adequacy during POEM. Techniques such as the double‐scope method [6], submucosal injection of indocyanine green into the gastric lesser curvature to confirm tunnel extension [7], and fluoroscopy with a distal clip marker [8] are mainly used to confirm that the submucosal tunnel has sufficiently extended beyond the esophagogastric junction into the gastric side, typically by 2–3 cm. In contrast, the EndoFLIP system has been used to assess esophagogastric junction distensibility during POEM [9, 10, 11]. Many centers perform POEM while confirming the distal extent of the myotomy using these objective modalities. However, the availability of such tools varies across institutions, and some centers rely primarily on endoscopic appearance to judge the adequacy of gastric‐side myotomy. In clinical practice, however, we frequently observe that the endoscopic esophagogastric junction (EGJ) appears more open immediately after submucosal tunneling—even before myotomy is performed. We refer to this misleading appearance as pseudo‐dilation. This phenomenon may create the impression of lower esophageal sphincter (LES) relaxation based on luminal appearance, although myotomy has not yet been performed. Therefore, this study aimed to evaluate changes in EGJ appearance during POEM and to determine the frequency of pseudo‐dilation.

## 2 Methods

### 2.1 Study Design and Patients

This retrospective descriptive study included consecutive patients who underwent POEM at our institution between February 2025 and June 2025. Clinical data and procedure videos were retrospectively analyzed using prospectively collected electronic medical records. The study protocol was approved by the institutional ethics committee (2025‐0516). Eligible patients for POEM were diagnosed using upper gastrointestinal endoscopy, high‐resolution manometry, and esophagography. Patients with prior endoscopic or surgical interventions, including pneumatic dilation or Heller–Dor myotomy, were excluded.

### 2.2 POEM Procedure

For patients receiving antithrombotic treatment, periprocedural antithrombotic agents were managed according to the Japan Gastroenterological Endoscopy Society guidelines [12, 13]. All procedures were performed under general anesthesia using a GIF‐H290T endoscope (Olympus, Tokyo, Japan) equipped with a short ST hood (DH‐28GR; Fujifilm, Tokyo, Japan). A second endoscope (GIF‐1200N; Olympus) was used for the double‐scope method. Submucosal injection using saline mixed with indigo carmine and tunnel creation were performed using a TriangleTip Knife J (Olympus Medical Systems) with a hood attachment [14]. An electrosurgical generator (VIO3; Erbe, Germany) was used with the following settings: EndoCut I mode (Effect 3, Cut duration 2, Cut interval 2) and spray coagulation at 4.0. Submucosal tunneling was advanced across the EGJ, and the distal extent of the tunnel was confirmed using the double‐scope method with a GIF‐1200N endoscope (Olympus, Tokyo, Japan). Additional tunneling was performed when the submucosal tunnel had not sufficiently extended 2–3 cm beyond the esophagogastric junction into the gastric side. Selective myotomy of the circular muscle was then completed. The gastric‐side myotomy was typically extended approximately three centimeters beyond the esophagogastric junction. The mucosal entry was closed using a clip (EZ Clip; Olympus Co., Tokyo, Japan).

### 2.3 Double‐Scope Method

The double‐scope method was used to confirm that the submucosal tunnel had sufficiently extended 2–3 cm beyond the esophagogastric junction into the gastric side during POEM [6]. In this technique, two endoscopes were introduced transorally. One endoscope was advanced into the submucosal tunnel, and its tip was visualized using transillumination at the tunnel endpoint. The second endoscope, advanced into the gastric lumen, confirmed the presence of transillumination to ensure adequate extension of the submucosal tunnel beyond the EGJ.

### 2.4 Data Collection

The following variables were collected: patient characteristics (age, sex, and disease duration), Eckardt score [15], procedure time, gastric‐side myotomy length, and adverse events. Procedure time was defined as the duration from mucosal incision to mucosal closure. Gastric‐side myotomy length was measured endoscopically from the squamocolumnar junction (SCJ) toward the gastric side. Adverse events included delayed bleeding and gastrointestinal perforation (including mucosal injury). Data were extracted from electronic medical records.

### 2.5 Definition of Endoscopic Junctional Dilation and Pseudo‐dilation

**FIGURE 1:**
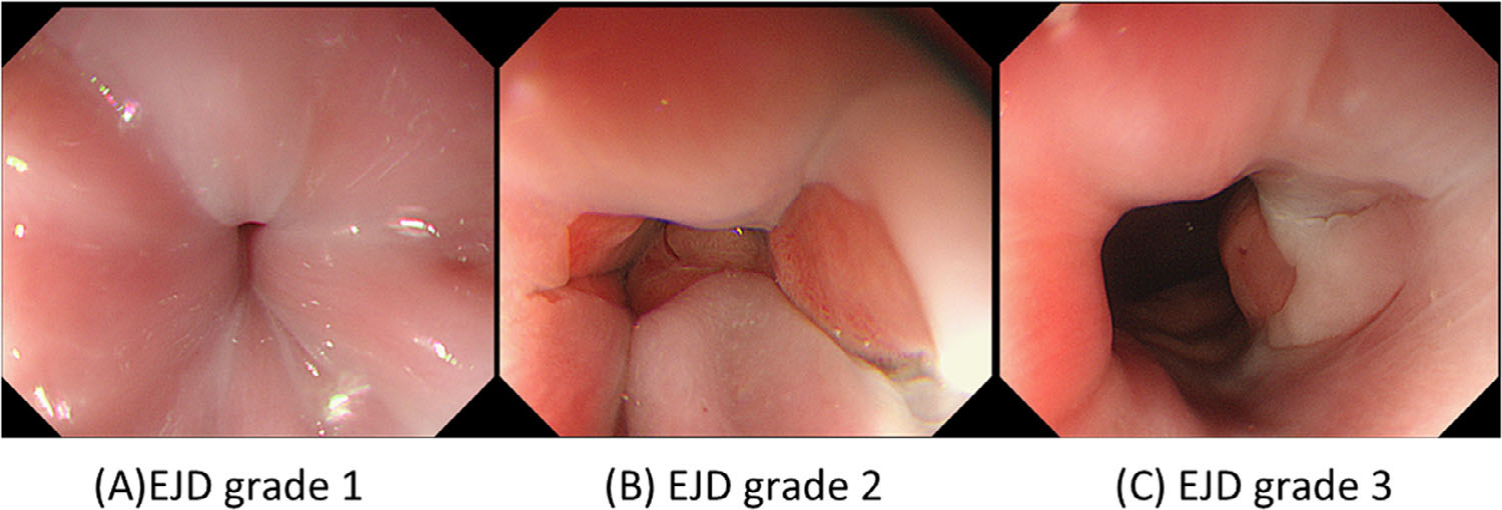
Representative endoscopic images of the Endoscopic Junctional Dilation (EJD) grades. (A) EJD Grade 1: A clear rosette sign is observed, and neither the gastric mucosa nor the gastric lumen is visible from the esophageal side. (B) EJD Grade 2: The squamocolumnar junction (SCJ) becomes visible, while the gastric lumen remains unseen. (C) EJD Grade 3: Both the gastric mucosa and gastric lumen are clearly visible from the esophageal side, giving the appearance of a marked lower esophageal sphincter (LES) opening.

The Endoscopic Junctional Dilation Grade (EJD grade) was newly established in this study to describe the apparent opening of the esophagogastric junction based solely on endoscopic appearance. This three‐level scale classifies the visual impression of LES relaxation as follows. Grade 1 corresponds to the presence of a clear rosette sign, with no visualization of the gastric mucosa or gastric lumen from the esophageal side. Grade 2 is defined by visualization of the SCJ, while the gastric lumen remains unseen. Grade 3 represents the stage at which both the gastric mucosa and gastric lumen become clearly visible from the esophageal side. Representative endoscopic images will be provided in Figure 1. Pseudo‐dilation was defined as an increase in EJD grade from Grade 1 before POEM to Grade 2 or 3 immediately after submucosal tunneling, prior to any myotomy. The phenomenon is demonstrated in Video S1.

### 2.6 Outcome Measures

EGJ opening was evaluated using the EJD grade at three predefined stages during the procedure: before POEM, after submucosal tunneling, and after myotomy. All endoscopic images obtained at these three time points were independently assessed by two certified endoscopists. One was an expert endoscopist who had performed more than 100 POEM procedures, and the other was a trainee who had performed more than 20 POEM procedures. Clinical success was defined as an Eckardt score of ≤ 3 at 2 months after the procedure.

### 2.7 Statistical Analysis

Categorical variables were summarized as frequencies and percentages, whereas continuous variables were presented as mean ± standard deviation or median with range. Given that the EJD grade is an ordinal variable assessed repeatedly within the same subjects across the three procedural stages, differences were primarily evaluated using the Friedman test, a non‐parametric method appropriate for detecting the overall differences in repeated ordinal measures. When overall significance was observed, post hoc pairwise comparisons were conducted using the Wilcoxon signed‐rank test with Bonferroni correction to account for multiple comparisons.

In addition, the Bickenböller statistic was used to assess transitions in EJD grade and to evaluate marginal homogeneity of grade distributions across the three procedural stages. This approach was included to complement the Friedman analysis by examining whether observed changes reflected meaningful shifts in the overall distribution of EJD grades, rather than only detecting ordinal rank differences.

The combined use of these methods allowed for a more comprehensive assessment of intraprocedural changes, capturing both statistical differences in ordinal ranking (Friedman test) and the extent of distributional changes (Bickenböller statistic), thereby providing a more clinically interpretable evaluation of EGJ opening during POEM.

Additionally, to appropriately evaluate the generalizability of the present findings to broader clinical practice, interobserver agreement for the EJD grade [1, 2, 3] was evaluated using Cohen's κ and quadratic weighted κ statistics. The strength of agreement was interpreted according to the Landis and Koch criteria [16]. Statistical analyses were performed using Stata version 14.0, with *p* < 0.05 considered to be significant.

## 3 Results

### 3.1 Patient Characteristics

A total of 65 patients underwent POEM. Patient characteristics are summarized in Table 1. The median age was 49 years (range, 20–92), and 31 patients (47.7%) were male. The median BMI was 20.9 (range, 14.8–38.2). The median American Society of Anesthesiologists physical status was 1 (range, 1–3). The median disease duration was 30 months (range, 2–524). Based on the Chicago classification, 36 patients (55.4%) were Type I, 24 (36.9%) were Type II, and five (7.7%) had esophagogastric junction outflow obstruction. Radiographic classification of esophageal achalasia showed a straight type in 62 patients (95.4%) and a sigmoid type in three patients (4.6%). The median integrated relaxation pressure was 22.85 mmHg (range, −1.8–103.5).

**TABLE 1:** Baseline patient characteristics (*n* = 65).

Variable	Value
Age, median (range), years	49 (20–92)
Male sex, *n* (%)	31 (47.7%)
BMI, median (range)	20.9 (14.8–38.2)
ASA physical status, median (range)	1 (1–3)
Disease duration, median (range), months	30 (2–524)
Chicago classification, *n* (%)	
Type I	36 (55.4%)
Type II	24 (36.9%)
EGJOO	5 (7.7%)
Radiographic classification of esophageal achalasia, *n* (%)	
Straight type, Sigmoid type	62 (95.4%), 3 (4.6%)
IRP, median (mmHg)	22.85 (‐1.8–103.5)

Abbreviations: ASA, American Society of Anesthesiologists; BMI, body mass index; EGJOO, esophagogastric junction outflow obstruction; IRP, integrated relaxation pressure.

### 3.2 Procedural Outcomes

Procedural outcomes are presented in Table 2. The median procedure time was 80 min (range, 38–134). The median total myotomy length was 7 cm (range, 5–10), and the median gastric‐side myotomy length was 3 cm (range, 2–5). All procedures were performed using the anterior approach. One intraoperative mucosal injury occurred and was treated endoscopically. The median Eckardt score improved from 6 (range, 1–11) preoperatively to 1 (range, 1–4) after POEM. Clinical success, defined as an Eckardt score of ≤ 3 at 2 months, was achieved in 64 of 65 patients (98.5%).

**TABLE 2:** Procedural outcomes (*n* = 65).

Variable	Value
Procedure time, median (range), minutes	80 (38–134)
Total myotomy length, median (range), cm	7 (5–10)
Gastric‐side myotomy length, median (range), cm	3 (2–5)
Approach	
Anterior approach, *n* (%)	65 (100%)
Intraoperative events	
Mucosal injury, *n* (%)	1 (1.5%)
Eckardt score, median (range)	
Preoperative	6 (1–11)
Postoperative	1 (1–4)
Clinical success rates, *n* (%)	64 (98.5%)

### 3.3 Distribution of EJD Grade at Each Procedural Stage

The distribution of EJD grades at each procedural stage is summarized in Table 3. Before POEM, nearly all patients exhibited tight closure of the EGJ, with EJD Grade 1 in 64 of 65 patients (98.5%) and Grade 2 in only one patient (1.5%). After submucosal tunneling, the distribution shifted markedly toward higher grades, with Grade 2 observed in 49 patients (75.4%) and Grade 3 in 14 patients (21.5%). Following myotomy, no patients remained in Grade 1, while Grade 2 and Grade 3 were observed in 43 patients (66.2%) and 22 patients (33.8%), respectively. Overall, pseudo‐dilation occurred in 62 of 65 patients (95.4%).

**TABLE 3:** Distribution of Endoscopic Junctional Dilation (EJD) grades at each procedural stage.

		Before POEM (*n* = 65)	After submucosal tunneling (*n* = 65)	After myotomy (*n* = 65)
EJD Grade	Grade 1	64 (98.5%)	2 (3.1%)	0 (0%)
	Grade 2	1 (1.5%)	49 (75.4%)	43 (66.2%)
	Grade 3	0 (0%)	14 (21.5%)	22 (33.8%)

Values are presented as the number of patients (%).

### 3.4 Statistical Comparison of EJD Grades Across Procedural Stages

The Friedman test demonstrated a significant difference in EJD grades across the three procedural stages (*X* ^2^ = 46.95; *p* < 0.001). Post hoc pairwise comparisons using the Wilcoxon signed‐rank test with Bonferroni correction showed significant differences between the pre‐POEM and post‐tunneling stages and between pre‐POEM and post‐myotomy stages (both *p* < 0.001). A statistically significant difference was also observed between the post‐tunneling and post‐myotomy stages (*p* = 0.002).

**FIGURE 2:**
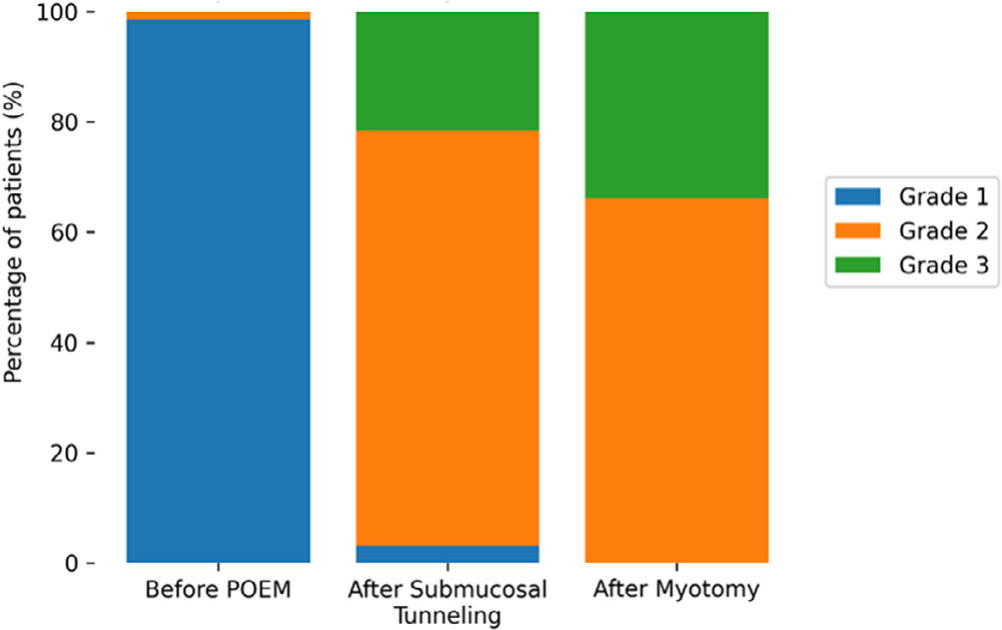
Distribution of the Endoscopic Junctional Dilation (EJD) grades across procedural stages. Stacked bar charts show the proportion of patients with EJD grades 1, 2, and 3 before peroral endoscopic myotomy (POEM), after submucosal tunneling, and after myotomy. A marked shift from grade 1 to grades 2–3 occurs immediately after tunneling, while limited additional change is observed following myotomy.

Despite this, the magnitude of change between the post‐tunneling and post‐myotomy stages was limited, with the majority of patients showing no change in the EJD grade. Consistent with this finding, analysis using the Bickenböller statistic demonstrated no significant difference between these two stages (*p* = 0.124), indicating that the overall distribution of EJD grades remained unchanged. These distributional patterns corresponding to the statistical findings are illustrated in Figure 2.

### 3.5 Interobserver Agreement Among Endoscopists in Evaluating EJD

The interobserver agreement between the two endoscopists was perfect for the EJD grade before POEM (κ = 1.00, weighted κ = 1.00). The interobserver agreement for the EJD grade after submucosal tunneling showed substantial agreement (κ = 0.889, 95% confidence interval [CI] 0.78–0.99; weighted κ = 0.940, 95% CI 0.87–0.99). After myotomy, agreement remained high (κ = 0.923, 95% CI 0.85–0.99; weighted κ = 0.966, 95% CI 0.92–0.99).

## 4 Discussion

In this study, we assessed the endoscopic status of the EGJ in achalasia patients at three different time points during POEM: before POEM, after submucosal tunneling, and after myotomy. Apparent endoscopic opening of the EGJ occurred immediately after submucosal tunneling and is referred to as pseudo‐dilation.

Although functional changes of the esophagogastric junction during POEM have been evaluated using EndoFLIP [9, 10, 11], endoscopic changes in EGJ appearance immediately after submucosal tunneling have not been well characterized. To our knowledge, the present study is the first to assess the EGJ status after submucosal tunneling and before myotomy, and it revealed the presence of pseudo‐dilation. As a result, we found that this phenomenon occurred in as many as 95.4% of POEM patients. Pseudo‐dilation represents an apparent endoscopic opening of the EGJ without preceding myotomy and does not necessarily reflect functional relaxation of the LES. Previous studies using EndoFLIP have demonstrated that esophagogastric junction distensibility does not increase, and may even decrease, following submucosal tunneling [11]. These findings reinforce that apparent endoscopic opening does not necessarily correspond to true physiological relaxation of the EGJ. Endoscopic appearance alone may not reliably distinguish the procedural stage of POEM, as apparent luminal opening is frequently observed even before myotomy. Therefore, reliance solely on endoscopic findings may be limited when assessing whether sufficient gastric‐side myotomy has been achieved. Although objective intraoperative assessment methods, such as the double‐scope technique, are generally used to confirm whether the submucosal tunnel has adequately extended into the gastric side beyond the esophagogastric junction, such methods may not always be feasible depending on institutional resources. In such settings, awareness of pseudo‐dilation may be important when interpreting luminal opening during POEM. In the present study, pseudo‐dilation was frequently observed after submucosal tunneling; however, the exact timing of this phenomenon during the procedure was not evaluated. Therefore, even when a luminal opening is observed endoscopically, it does not necessarily indicate that the submucosal tunnel has sufficiently extended into the gastric side beyond the esophagogastric junction. Further studies with a larger sample size are needed to clarify the generalizability of these findings.

Several possible explanations may account for the apparent widening of the EGJ after submucosal tunneling. One potential factor is thermal influence from electrocautery during tunneling, which may transiently alter tissue configuration and neuromuscular integrity around the EGJ [17]. Mechanical effects related to repeated endoscope passage across the LES may also contribute to the altered appearance [18]. Although mechanical stimulation associated with the passage of multiple endoscopes, such as in the double‐scope technique, may theoretically contribute to transient dilation of the EGJ, the EJD grade in the present study was evaluated before performing the double‐scope method. Therefore, pseudo‐dilation was unlikely to be primarily caused by mechanical dilation related to the passage of multiple endoscopes. These explanations remain speculative, and further studies are required to clarify the true mechanism of pseudo‐dilation.

The EJD grading system was designed as a descriptive classification to capture stepwise changes in endoscopic appearance during POEM; thus, the clinical significance in each grade remains unclear. However, this EJD grading system appears reproducible across endoscopists with different levels of experience. In our study, high interobserver agreement was observed between two endoscopists with different levels of experience in POEM, suggesting that the EJD grade may be a reproducible measure of intraprocedural endoscopic change. Further studies are needed to clarify the clinical significance of this grading system, including its association with symptoms, procedural outcomes, and long‐term therapeutic efficacy.

This study has several limitations. First, this was a retrospective single‐center study conducted at a Japanese tertiary care center. Second, no a priori sample size calculation was performed due to the retrospective nature of the study; however, the observed changes in EJD grade were large and consistent across patients, with nearly all cases demonstrating an increase in grade following submucosal tunneling. These findings suggest clinically meaningful changes, although the adequacy of the sample size cannot be formally confirmed. Third, all procedures involved a standard gastric‐side myotomy of approximately 3 cm, limiting evaluation in cases with shorter or insufficient myotomies. Fourth, the EJD grade was assessed exclusively through endoscopic appearance, without physiologic validation, because EndoFLIP is not approved for clinical use in Japan. Fifth, the two endoscopists who evaluated the images partially overlapped with the operators who performed the procedures. Image assessment was conducted independently; however, blinding to procedural details and clinical outcomes was not applied. Finally, the long‐term clinical implications of pseudo‐dilation remain uncertain and warrant further study.

## 5 Conclusion

This study demonstrated the presence of pseudo‐dilation during POEM. Further studies are needed to confirm the reproducibility of pseudo‐dilation across institutions and its impact on procedural decision‐making in POEM.

## Author Contributions

**Kei Ushikubo** contributed to conceptualization, methodology, data curation, formal analysis, and writing – original draft. **Haruhiro Inoue** contributed to conceptualization, study design, investigation, supervision, and writing – review & editing. **Ippei Tanaka** and **Kohei Shigeta** contributed to conceptualization, study design, investigation, supervision, and writing – review & editing. **Nikko Raymundo** contributed to formal analysis, investigation, and language editing. **Kazuki Yamamoto**, **Yuta Tamaru**, **Satoshi Abiko**, **Yohei Nishikawa**, and **Mayo Tanabe** contributed to investigation and writing – review & editing. **Manabu Onimaru**, **Koji Otuka**, **Takayoshi Ito**, and **Noboru Yokoyama** contributed to supervision and writing – review & editing. All authors approved the final manuscript.

## Funding

This study received no specific funding. The authors have nothing to report.

## Ethics Statement

This study was approved by the Institutional Ethics Committee of Showa Medical University (Approval No. 2025‐0516).

## Consent

Informed consent was obtained using an opt‐out approach.

## Conflicts of Interest

The authors declare no conflicts of interest.

## Supporting information




**VIDEO S1 Endoscopic demonstration of pseudo‐dilation: early EGJ opening before myotomy**. This video shows early EGJ opening before myotomy—a misleading appearance we refer to as pseudo‐dilation. EGJ: esophagogastric junction.

## References

[1] H. Inoue , H. Minami , Y. Kobayashi , et al., “Peroral Endoscopic Myotomy (POEM) for Esophageal Achalasia,” Endoscopy 42 (2010): 265–271. 20354937 10.1055/s-0029-1244080

[2] G. E. Boeckxstaens , G. Zaninotto , and J. E. Richter , “Achalasia,” Lancet 383 (2014): 83–93. 23871090 10.1016/S0140-6736(13)60651-0

[3] H. Inoue , K. M. Tianle , H. Ikeda , et al., “Peroral Endoscopic Myotomy for Esophageal Achalasia: Technique, Indication, and Outcomes,” Thoracic Surgery Clinics 21 (2011): 519–525. 22040634 10.1016/j.thorsurg.2011.08.005

[4] H. Inoue , H. Sato , H. Ikeda , et al., “Per‐oral Endoscopic Myotomy: A Series of 500 Patients,” Journal of the American College of Surgeons 221 (2015): 256–264. 26206634 10.1016/j.jamcollsurg.2015.03.057

[5] L. Quénéhervé , B. Vauquelin , A. Berger , et al., “Risk Factors for Clinical Failure of Peroral Endoscopic Myotomy in Achalasia,” Frontiers in Medicine 9 (2022): 1099533. 36569161 10.3389/fmed.2022.1099533 PMC9773253

[6] K. L. Grimes , H. Inoue , M. Onimaru , et al., “Double‐scope per Oral Endoscopic Myotomy (POEM): A Prospective Randomized Controlled Trial,” Surgical Endoscopy 30 (2016): 1344–1351. 26173548 10.1007/s00464-015-4396-2

[7] H. Minami , H. Inoue , A. Haji , et al., “Per‐oral Endoscopic Myotomy: Emerging Indications and Evolving Techniques,” Digestive Endoscopy 27, no. 2 (2015): 175–181. 25040806 10.1111/den.12328

[8] V. Kumbhari , S. Besharati , A. Abdelgelil , et al., “Intraprocedural Fluoroscopy to Determine the Extent of the Cardiomyotomy During Per‐oral Endoscopic Myotomy (With video),” Gastrointestinal Endoscopy 81 (2015): 1451–1456. 25887723 10.1016/j.gie.2015.01.052

[9] J. Chang , I. K. Yoo , S. Günay , et al., “Clinical Usefulness of Esophagogastric Junction Distensibility Measurement in Patients With Achalasia Before and After Peroral Endoscopic Myotomy,” Turkish Journal of Gastroenterology 31 (2020): 362–367. 10.5152/tjg.2020.19105 PMC7289165 32519955

[10] Y. Fujiyoshi , M. R. A. Fujiyoshi , K. Khalaf , et al., “Association of Gastric Myotomy Length in Peroral Endoscopic Myotomy (POEM) With Gastro‐esophageal Junction Distensibility Measured by Endoluminal Functional Lumen Imaging Probe (EndoFLIP),” Esophagus 21 (2024): 563–570. 39186141 10.1007/s10388-024-01081-9

[11] T. B. Knowles , A. S. Jackson , S. C. Chang , et al., “Changes in Distensibility Index During an Incremental POEM Myotomy,” Journal of Gastrointestinal Surgery 26 (2022): 1140–1146. 35233701 10.1007/s11605-022-05278-0

[12] M. Kato , N. Uedo , S. Hokimoto , et al., “Guidelines for Gastroenterological Endoscopy in Patients Undergoing Antithrombotic Treatment: 2017 Appendix on Anticoagulants Including Direct Oral Anticoagulants,” Digestive Endoscopy 30 (2018): 433–440. 29733468 10.1111/den.13184

[13] K. Fujimoto , M. Fujishiro , M. Kato , et al., “Guidelines for Gastroenterological Endoscopy in Patients Undergoing Antithrombotic Treatment,” Digestive Endoscopy 26 (2014): 1–14. 10.1111/den.12183 24215155

[14] H. Inoue , Y. Kimoto , M. J. Navarro , et al., “Triangle‐tip Jet Knife With Hood Attachment: Novel Modification to Endoscopic Knife,” VideoGIE 8 (2023): 383–384. 37849777 10.1016/j.vgie.2023.06.018 PMC10577583

[15] V. F. Eckardt , C. Aignherr , and G. Bernhard , “Predictors of Outcome in Patients With Achalasia Treated by Pneumatic Dilation,” Gastroenterology 103 (1992): 1732–1738. 1451966 10.1016/0016-5085(92)91428-7

[16] J. R. Landis and G. G. Koch , “The Measurement of Observer Agreement for Categorical Data,” Biometrics 33 (1977): 159–174. 843571

[17] T. Burdyga and S. Wray , “On the Mechanisms Whereby Temperature Affects Excitation–Contraction Coupling in Smooth Muscle,” Journal of General Physiology 119 (2002): 93–104. 11773241 10.1085/jgp.119.1.93 PMC2233859

[18] Y. Jiang , V. Bhargava , and R. K. Mittal , “Mechanism of Stretch‐activated Excitatory and Inhibitory Responses in the Lower Esophageal Sphincter,” American Journal of Physiology Gastrointestinal and Liver Physiology 297, no. 2 (2009): G397–405. 19520741 10.1152/ajpgi.00108.2009 PMC2724084

